# Twist of Fate: Diagnosing and Managing Gallbladder Volvulus in an Elderly Patient

**DOI:** 10.7759/cureus.78813

**Published:** 2025-02-10

**Authors:** Ali Wuheb, Mohamed Ismaiel, Hassan Abdulrahman, Parth Gada, Ayesha Ragavoodoo, Mehvish Alavi, Prem Thambi

**Affiliations:** 1 General Surgery, James Cook University Hospital, Middlesbrough, GBR; 2 Radiology, James Cook University Hospital, Middlesbrough, GBR; 3 Colorectal Surgery, James Cook University Hospital, Middlesbrough, GBR

**Keywords:** gallbladder volvulus, gallstone disease (gsd), gangrenous cholecystitis, laparoscopic cholecystectomy, right upper quadrant abdominal pain

## Abstract

Gallbladder volvulus (GV) is a rare surgical emergency characterized by the twisting of the gallbladder around its mesentery, leading to vascular compromise and gangrene. It is often misdiagnosed as acute gangrenous cholecystitis due to overlapping symptoms, making preoperative diagnosis challenging. Definitive identification is typically made intraoperatively. Contributory factors include advanced age, female gender, low body mass index, and increased gallbladder mobility. Urgent surgical intervention is crucial to prevent severe complications such as perforation and biliary peritonitis. We report a case of an elderly woman with a low BMI who had a history of right hemicolectomy for locally advanced cecal cancer and ongoing immunotherapy for pelvic recurrence. She presented with acute epigastric pain, nausea, and vomiting but no fever or jaundice. Examination revealed a tender upper abdomen with a palpable mass. Laboratory investigations showed leucocytosis with unremarkable CRP, liver, and renal function tests. A CT scan demonstrated a grossly distended midline gallbladder with a swirl sign at the cystic pedicle, consistent with GV. Supportive management was given, followed by urgent laparoscopic cholecystectomy. Intraoperatively, a gangrenous gallbladder twisted 360° twice around its mesentery was identified and safely removed without complications. The patient made an uneventful recovery and was discharged within 48 hours. This case underscores the diagnostic challenges of GV and highlights the importance of a high index of suspicion, particularly in elderly, frail patients with risk factors. Imaging findings such as the "swirl sign" and midline crossing of the gallbladder on CT scan aid preoperative diagnosis. Urgent surgical intervention remains the cornerstone of management, as delayed treatment can result in significant morbidity.

## Introduction

Gallbladder volvulus (GV), a rare surgical emergency, occurs when the gallbladder (GB) twists around its mesentery, obstructing its blood supply and potentially leading to gangrene [1]. This condition, first documented by Wendel in 1898, is also described as the "floating gallbladder" [2].

It remains difficult to diagnose it preoperatively and is often mistaken for acute cholecystitis due to its overlapping symptoms [3]. Additionally, imaging, including CT and ultrasound, often shows only distension and thickening of the gallbladder wall, findings that are also consistent with cholecystitis [2]. It is predominantly diagnosed intraoperatively when viewing the twisted pedicle of the gallbladder, with less than 10% accurately identified before surgery [1].

Several anatomical and demographic factors contribute to GV, including advanced age, female gender, weight loss, and liver atrophy, which increase gallbladder mobility and susceptibility to torsion [2]. Surgical intervention through cholecystectomy is the definitive treatment, with prompt management being crucial to avoid complications like gangrene, perforation, and biliary peritonitis [1].​ This case report explores a rare presentation of GV in an 85-year-old female, highlighting diagnostic challenges and imaging findings, and emphasizing the need for a high index of suspicion to ensure timely intervention.

## Case presentation

We present a case of an 85-year-old woman weighing 40 kg with a BMI of 15 and a surgical history that included a right hemicolectomy for locally advanced cecal cancer six years ago. She subsequently developed a pelvic recurrence, was treated with radiotherapy, and is currently on immunotherapy.

She presented to the emergency department with severe epigastric pain that had persisted for one day. She described the pain as squeezing in nature and unrelieved by simple analgesia. The pain was associated with nausea and vomiting, with no fever. While she had experienced similar pain for several weeks, it had intensified significantly on the day of her presentation. There were no features suggestive of bowel obstruction or jaundice.

**Figure 1:**
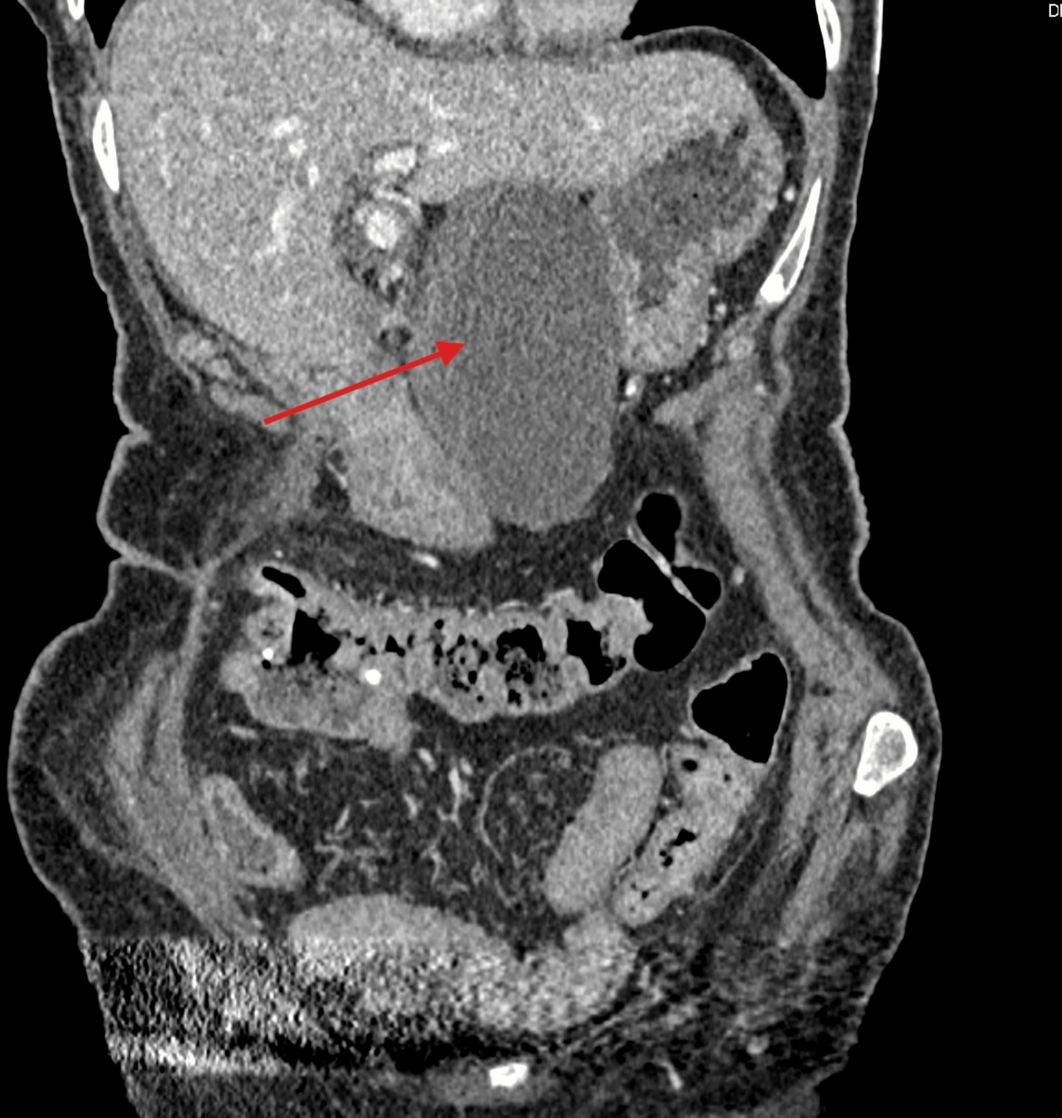
CT scan of the abdomen and pelvis showing a grossly distended gallbladder abnormally positioned in the midline (red arrow).

**Figure 2:**
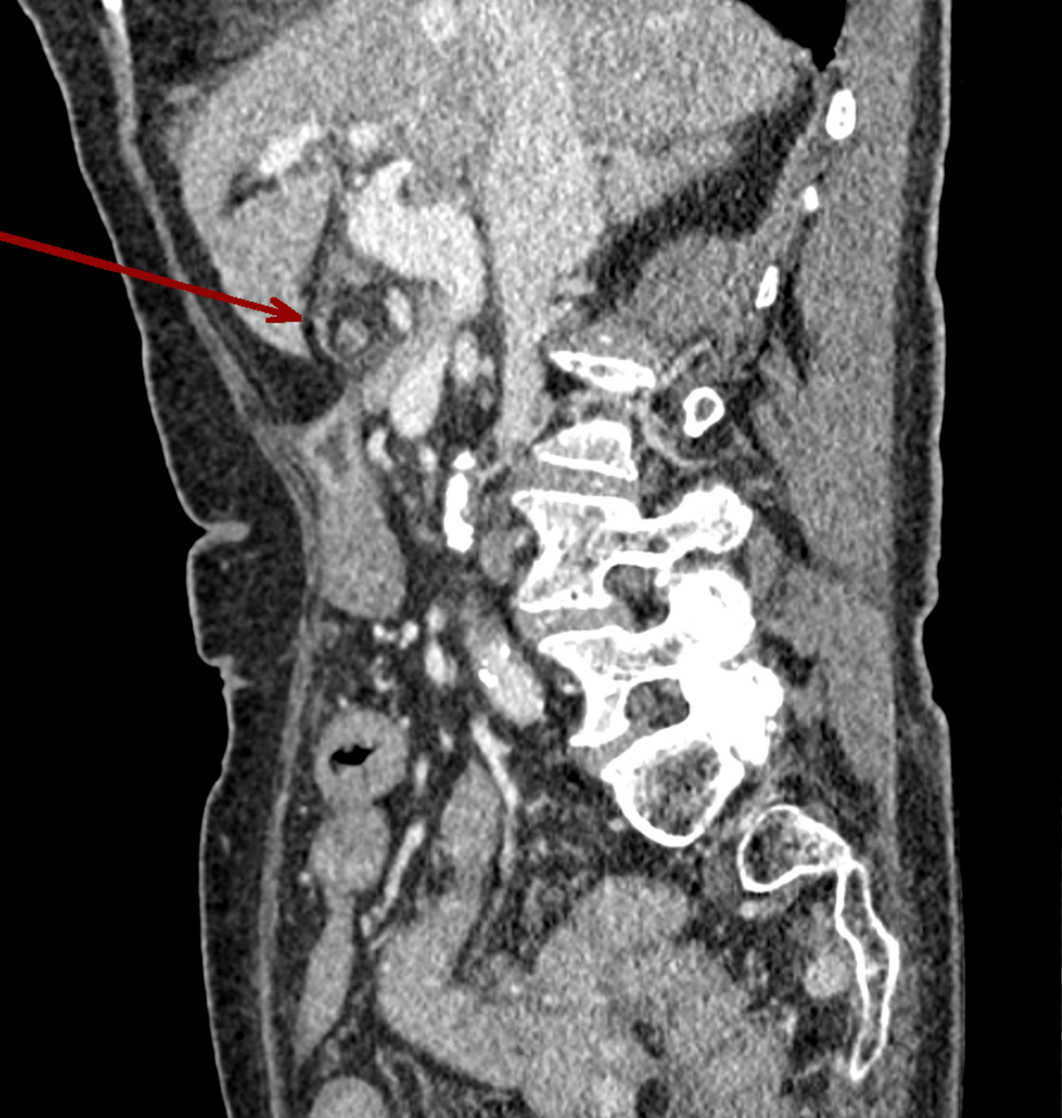
The sagittal view shows the twisted mesentery (red arrow).

**Figure 3:**
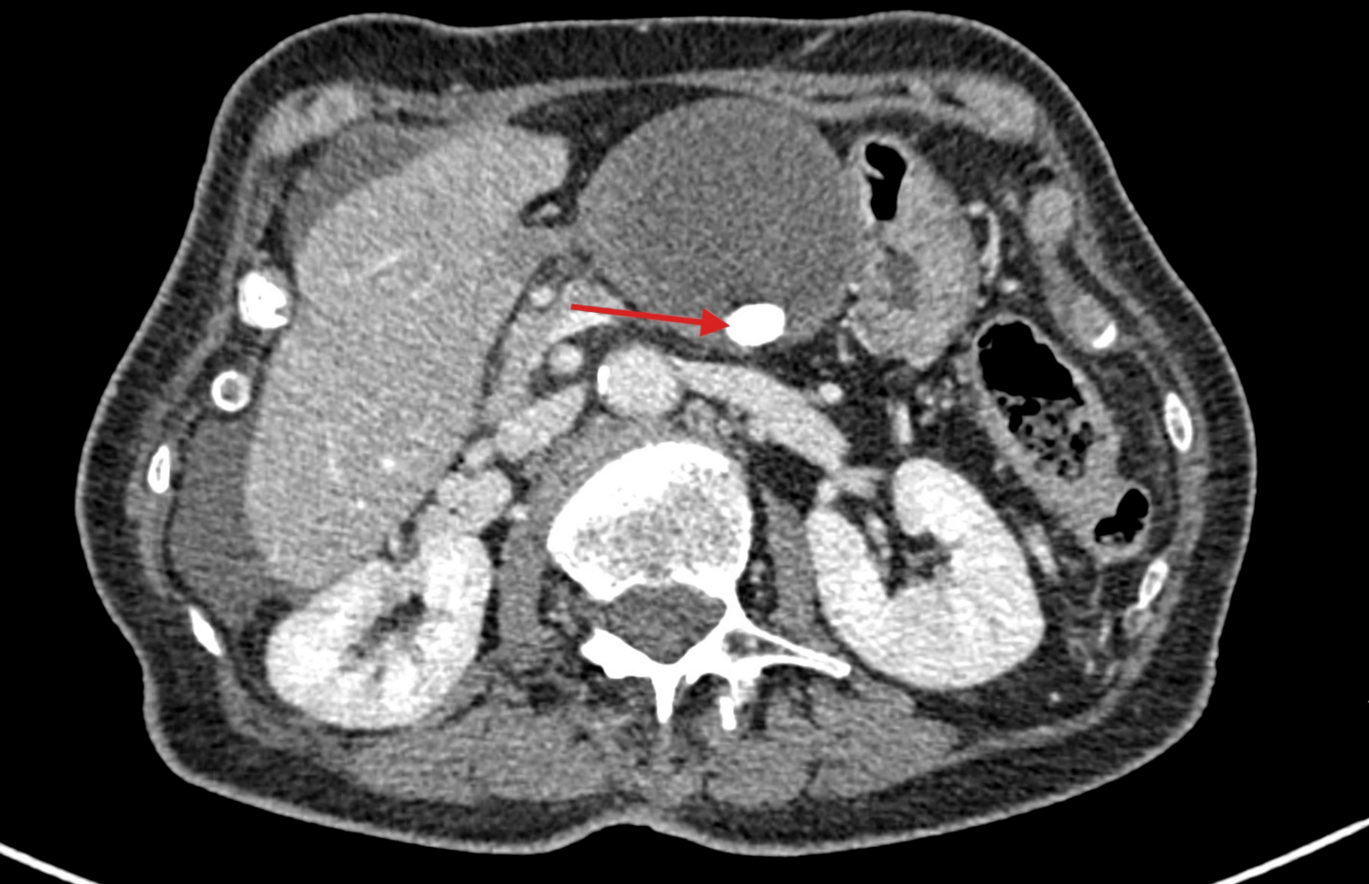
CT scan of the abdomen and pelvis revealed a grossly distended gallbladder with a 14-mm gallstone (red arrow).

On examination, her abdomen was tender in the right upper quadrant with a palpable mass that extended from the right upper quadrant to the epigastric area. Blood tests revealed a white blood cell count of 16 x 10^9^/L, a CRP of less than 5 mg/L, and normal liver and renal function tests. A CT scan of the abdomen and pelvis showed a grossly distended gallbladder that was abnormally positioned in the midline (Figure 1), outside its anatomical location, with a distinctive swirl sign at the pedicle of the cystic duct and artery (Figure 2), consistent with GV. The gallbladder wall appeared thickened with free fluid, extending around the subhepatic space and into the pelvis. Additionally, a 14-mm gallstone was noted (Figure 3).

Given the patient's age and her extensive ongoing surgical and cancer treatment history, we initially started supportive management to stabilize her condition with IV morphine, fluids, and antibiotics. After further evaluation by a consultant surgeon and an anesthesiologist, a decision was made to proceed with an urgent cholecystectomy.

**Figure 4:**
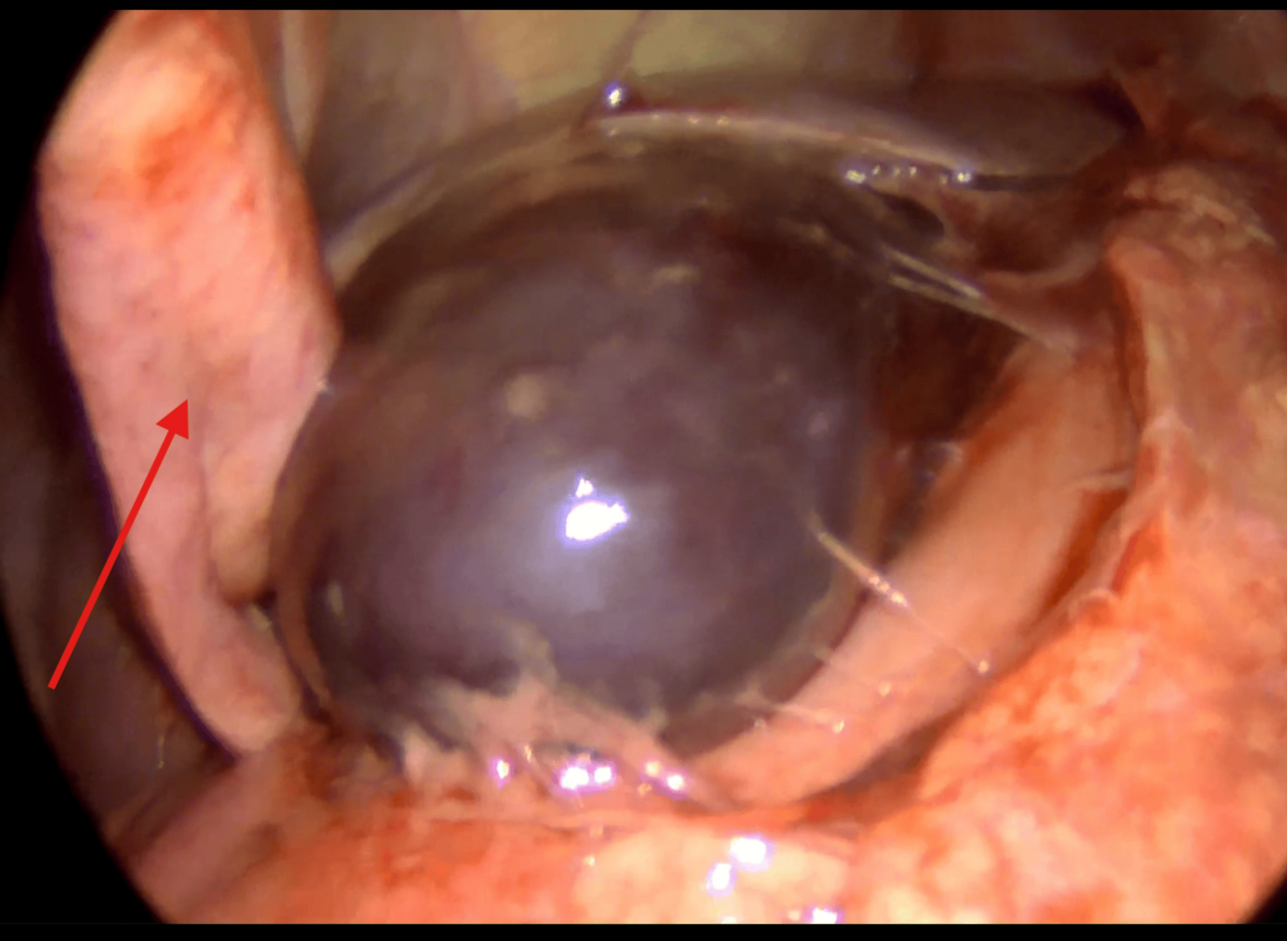
Intraoperative photography of a large gangrenous gallbladder crossing the midline, and the falciform ligament (red arrow) is pushed laterally to the gallbladder fundus.

**Figure 5:**
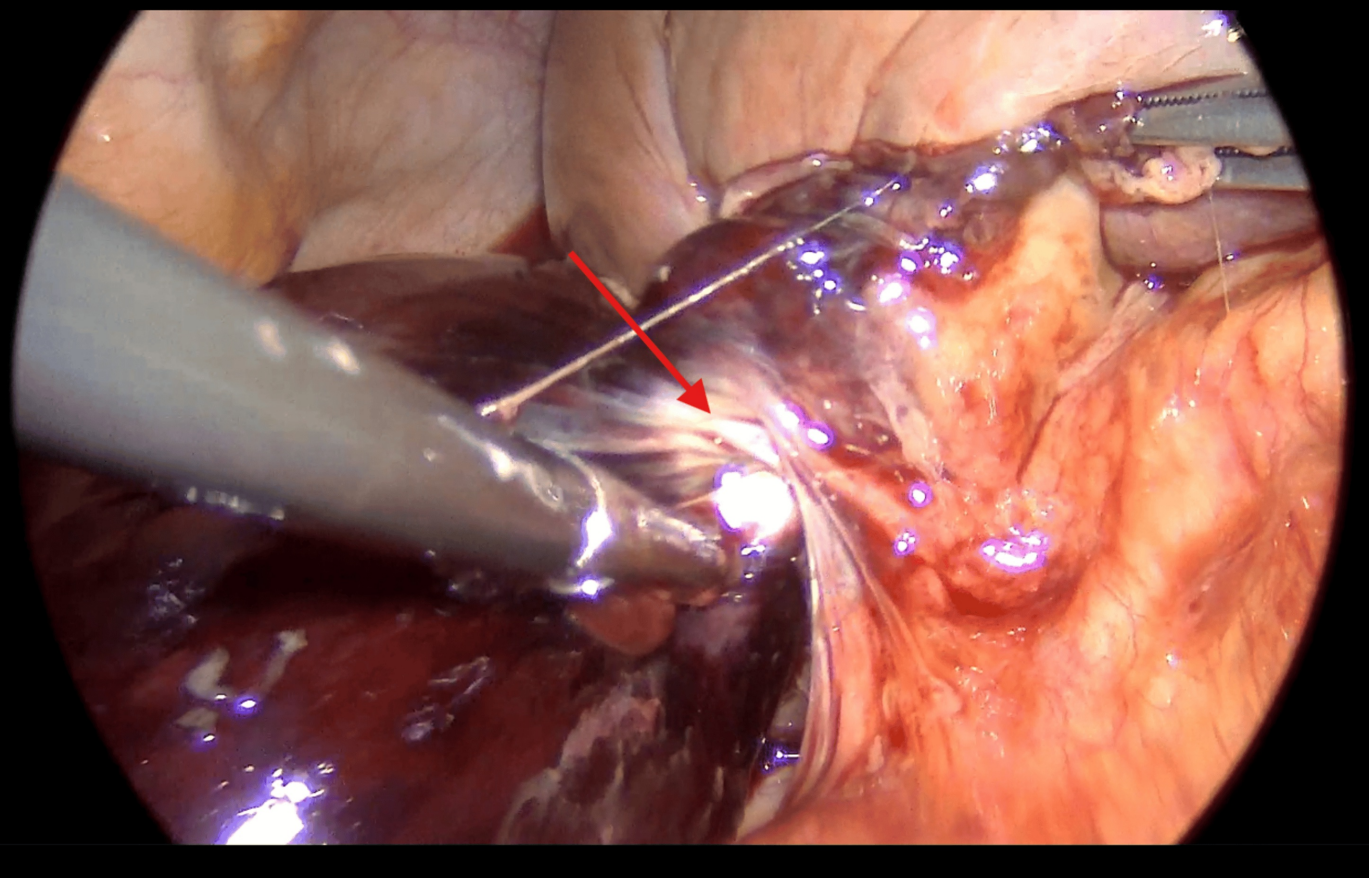
Intraoperative photography showing a large gangrenous gallbladder that twisted around its mesentery (red arrow).

**Figure 6:**
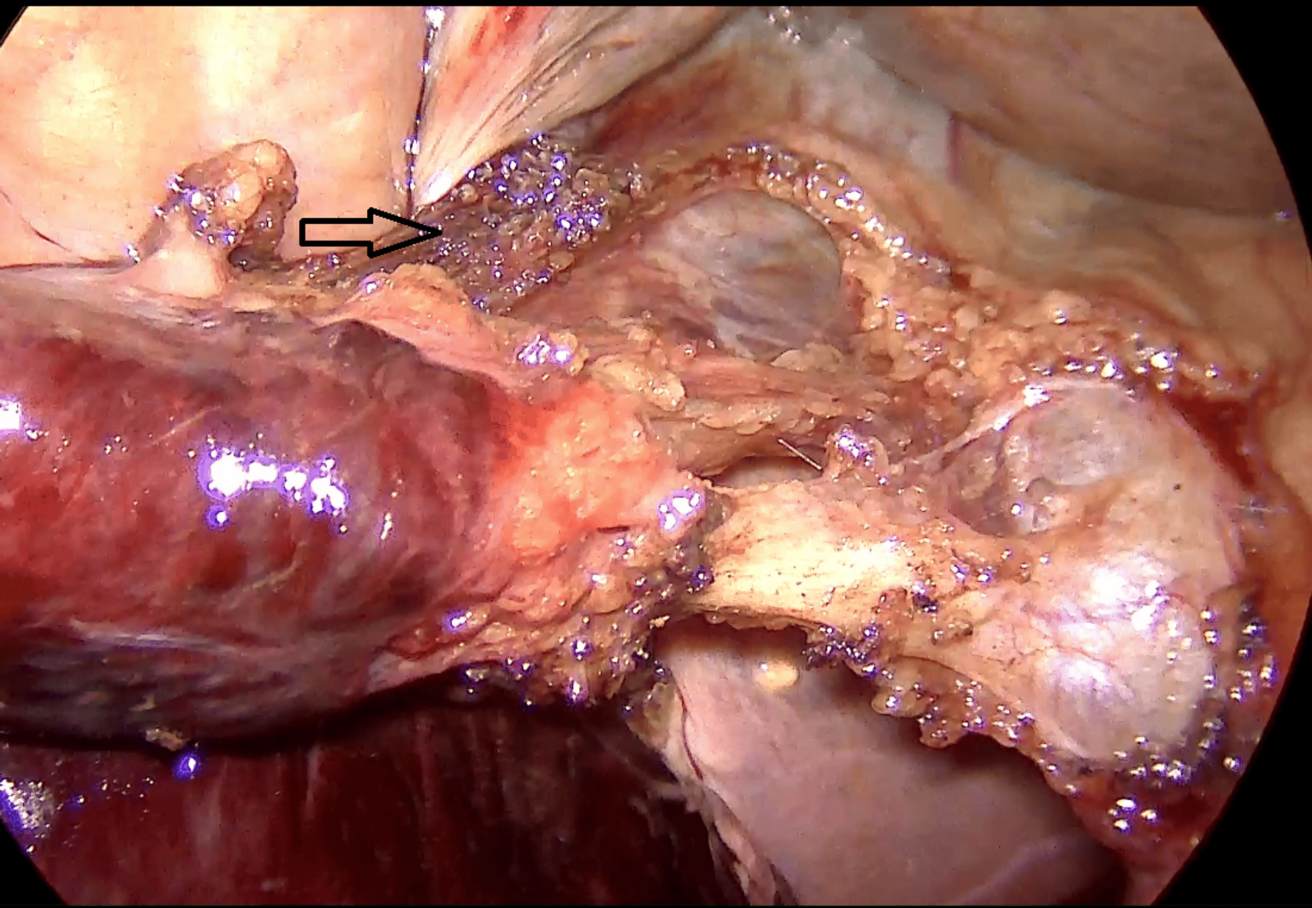
Intraoperative photography revealing the cystic duct and artery after careful dissection and minimal attachment of the gallbladder to the liver bed is noted before clip application (black arrow).

The anesthetic phase was uneventful, and a standard laparoscopic cholecystectomy technique was used. Intraoperative findings revealed a large gangrenous gallbladder that nearly crossed the midline, pushing the falciform ligament laterally (Figure 4), and twisted 360 degrees twice around its mesentery without perforating (Figure 5). Additionally, the gallbladder had a minimal attachment to the liver bed (Figure 6). The common bile duct appeared normal, and the gallbladder was successfully removed. The patient remained in the hospital for 48 hours postoperatively, during which her symptoms improved significantly. She was subsequently discharged home.

The patient was reviewed four weeks postoperation. She experienced a smooth recovery with good wound healing, and the histopathology report showed features suggestive of gangrenous cholecystitis secondary to volvulus with no evidence of malignancy.

## Discussion

GV is a rare but serious condition that can cause acute abdominal pain, yet it is not well-documented in surgical literature. To date, fewer than 400 cases have been reported, with an incidence of roughly one in 365,520 hospital admissions [2].

The causes, symptoms, and risk factors of GV have primarily been identified through case reports. A major diagnostic challenge is the condition's similarity to acute cholecystitis and other sources of upper abdominal pain. This overlap in symptoms often results in GV being misdiagnosed as acute cholecystitis.

The severity of GV is highlighted by its potential complications, such as gallbladder perforation, which can result in biliary peritonitis. Therefore, timely clinical suspicion is essential. GV should be considered in any case of upper abdominal pain, particularly in elderly females, where the condition is more prevalent.

While GV is commonly reported in elderly females, there have also been a few documented cases in younger patients [4] and even in children [5]. These cases highlight the variability in the age of presentation. In younger populations, GV is more prevalent in males, with a male-to-female ratio of 2:1. In contrast, in elderly patients aged 70 years and older, there is a higher prevalence in females, with a female-to-male ratio of 4:1 [1]. Our case involves an 85-year-old female patient, which aligns with this trend regarding gender and age presentation.

The exact etiology of GV is not fully understood, but several anatomical theories have been proposed. One suggestion is that a congenital abnormality in gallbladder attachment contributes to a hypermobile gallbladder. In these cases, the connection between the gallbladder and the liver may be partially or entirely absent, allowing the gallbladder to float freely within the peritoneal cavity [1].

According to the Gross classification, two anatomical variants predispose individuals to GV: (1) type A: a mesentery of variable length connects the liver to both the gallbladder and the cystic duct; (2) type B: a mesentery connects only the cystic duct to the liver [6,7].

Gallbladder torsion can also be categorized by the degree of rotation: (1) complete torsion: rotation exceeds 180°, preventing natural detorsion; (2) incomplete torsion: rotation is less than 180°, allowing for natural detorsion [7].

Both clockwise and counterclockwise rotations have been reported. Clockwise torsion is thought to arise from gastric and duodenal peristalsis, while counterclockwise torsion may be secondary to colonic peristalsis [8]. In our case, the gallbladder was attached only to the inferior surface of the liver, consistent with type A anatomy, and exhibited a 360° counterclockwise torsion that occurred twice. These findings align with those from Matías-García et al. [1], whose case involved an 87-year-old patient, suggesting that advanced age may be associated with an increased degree of torsion.

A low BMI is considered a significant risk factor for GV. Reduced pericholecystic fat, often resulting from significant weight loss, diminishes gallbladder support and increases its mobility [9]. Other contributing factors include liver atrophy, loss of visceral fat, reduced elasticity with aging, kyphoscoliosis, and arteriosclerosis with tortuous and rigid cystic arteries [6].

Furthermore, gallstones and acute cholecystitis are commonly found in GV cases, but they do not occur in every instance. Cases of GV without gallstones but with cholecystitis have been reported [1,8]. These findings suggest that in cases of GV, the cholecystitis may be primarily gangrenous in nature rather than gallstone-induced.

Preoperative diagnosis of GV remains a challenge, with only 10-26% of cases being diagnosed through clinical findings and advanced imaging techniques. Most diagnoses are confirmed intraoperatively [10].

Lau et al. [11] proposed the "Triad of Triads" to aid in identifying GV, comprising the following elements: clinical features: elderly patients, typically females, with a thin habitus and spinal deformity; symptoms: sudden onset of right upper quadrant pain accompanied by early emesis; physical findings: a non-toxic presentation, palpable right upper quadrant mass, and pulse-temperature discrepancy.

However, not all cases exhibit these criteria, which adds to the diagnostic complexity. This highlights the need for a high index of suspicion for GV in patients with atypical presentations.

Common radiographic features of GV, as described by Venkatesh et al. [12], include specific findings on ultrasound and CT imaging. Ultrasound may show an enlarged, floating gallbladder positioned outside its normal anatomical fossa. In contrast, CT imaging often reveals a massively distended gallbladder displaced from its usual position, with its orientation shifting from vertical to horizontal. Other characteristic signs observed on CT include the "swirl sign," caused by the indrawing of the vascular pedicle and surrounding fat, and the "bird beak sign," which indicates an abrupt tapering of the cystic duct. Additional radiological modalities, such as magnetic resonance cholangiopancreatography (MRCP) and hepatobiliary iminodiacetic acid (HIDA) scans, might reveal unique features like the "bull’s eye sign" or a fusiform common bile duct (CBD), aiding in the differentiation from other biliary pathologies [13].

In our case, the presence of the "swirl sign" on imaging was crucial in making a rare preoperative diagnosis of GV. Laparoscopic cholecystectomy is the primary treatment for GV and should be performed urgently to prevent complications such as perforation and biliary peritonitis. The intraoperative technique involves untwisting the volvulus and addressing any anatomical variations encountered. In our case, the gallbladder had crossed the midline and was pressing against the falciform ligament, which complicated access, especially through the epigastric port. This situation highlights the importance of tailoring port placement to ensure optimal visualization and access during surgery. Additionally, the hemoperitoneum in the subhepatic space required suction and irrigation before proceeding further. The absence of attachment between the gallbladder and the liver facilitated dissection and exposure of the redundant mesentery, a hallmark feature of GV. Despite these anatomical variations, the remaining steps of the procedure, including the clipping and ligation of the cystic duct and artery, followed the standard laparoscopic cholecystectomy protocol. Adapting the surgical approach to meet these unique challenges ensures safe and efficient management of GV while minimizing intraoperative risks.

## Conclusions

GV is a rare but significant cause of acute abdominal pain and is often misdiagnosed because its symptoms closely resemble those of acute cholecystitis. This case illustrates the critical role of radiological findings, particularly the "swirl sign," in making a rare preoperative diagnosis. The "swirl sign," which indicates twisting at the cystic pedicle, was crucial in identifying GV and distinguishing it from other biliary disorders. Recognizing this sign highlights the importance of advanced imaging techniques in facilitating timely and appropriate surgical intervention.

The observed 360° double twisting in our elderly patient suggests a potential link between advanced age and the severity of the torsion. Factors related to aging, such as decreased visceral fat, increased gallbladder mobility, and weakened supporting structures, may contribute to greater degrees of torsion. These findings align with current literature and underscore the necessity for heightened clinical and radiological vigilance in elderly patients presenting with atypical imaging features of upper abdominal pain. Laparoscopic cholecystectomy remains the mainstay of treatment and needs to be considered urgent as soon as the diagnosis of GV is clinically suspected or confirmed by radiology.
